# Just as Essential: The Mental Health of Educators During the COVID-19 Pandemic

**DOI:** 10.1017/dmp.2023.231

**Published:** 2024-01-18

**Authors:** Alyssa Schneider Carlson, Manny S. Stegall, Zoe Sirotiak, Felipe Herrmann, Emily B. K. Thomas

**Affiliations:** Department of Psychological and Brain Sciences, University of Iowa, Iowa City, IA, USA

**Keywords:** COVID-19, depression, anxiety, educators, teaching, psychological flexibility

## Abstract

**Objective:**

The coronavirus disease 2019 (COVID-19) pandemic deleteriously impacted physical and mental health. In the summer of 2020, return-to-learn plans were enacted, including virtual, hybrid, and in-person plans, impacting educators and students. We examined (1) how return-to-learn plan was related to depressive and social anxiety symptoms among educators and (2) how psychological flexibility related to symptoms.

**Methods:**

Educators (*N* = 853) completed a survey via Qualtrics that assessed internalizing symptoms, psychological flexibility, and occupational characteristics. Two one-way analyses of variance (ANOVAs) examined between-group differences in return-to-learn plans across depression and social anxiety. Two hierarchical linear regressions examined the relation between psychological flexibility components and depressive and social anxiety symptoms.

**Results:**

Median *T*-scores were well above the national normative means for General Depression (median *T*-score: 81) and Social Anxiety (median *T*-score: 67). There were no significant differences between reopening plans in general depression nor social anxiety *T*-scores. Psychological flexibility accounted for 33% of the variance in depressive symptoms and 24% of the variance in social anxiety symptoms.

**Conclusions:**

Results indicated high levels of psychiatric symptoms among educators during COVID-19, and psychological flexibility was associated with lower symptoms. Addressing educator mental health is of utmost importance in future research.

In December of 2019, the coronavirus disease 2019 (COVID-19) virus first emerged in the Wuhan province of China. Before long, the disease had spread throughout the world, causing physical illness and millions of deaths.^1^ For many, the pandemic brought with it not only physical health issues, but also psychiatric symptoms. Significantly higher rates of depression, anxiety, posttraumatic stress disorder (PTSD), suicidal ideation or behavior, substance use, and sleep difficulties have been found compared with prepandemic levels.^2,3^

Although adults around the world have experienced the deleterious effects of COVID-19, essential workers have been particularly vulnerable to stress, burnout, and psychiatric symptoms. Healthcare workers reported elevated rates of depression and anxiety.^4^ Among nursing staff, fatigue, depersonalization, and posttraumatic stress were reported.^5^ Physicians reported high levels of anxiety; moreover, mental exhaustion, fear of being infected, fear of infecting family members, and sleep difficulties were associated with anxiety.^6^ In a survey of health-care workers, nearly half endorsed moderate to severe symptoms of depression and anxiety.^7^

Although research regarding essential workers has focused on healthcare workers, educators also continued to work and faced risk of COVID-19 exposure. One study demonstrated a high proportion of educators reporting depression, anxiety, and stress, which was attributed, in part, to the need to adapt to different teaching modalities and adjust to the needs of the school district.^8^ Faced with the uncertainty of the pandemic, educators reported high levels of anxiety and depression, with female educators reporting greater anxiety symptoms.^9^ Educators faced declining well-being and quality of life, while also experiencing increased depression and anxiety.^10,11^

As schools were set to reopen in the fall semester of 2020, Johns Hopkins University launched a website that detailed nationwide return-to-learn plans that had been developed for grades K-12 and college across the United States (Johns Hopkins University, 2020; https://equityschoolplus.jhu.edu/reopening-policy-tracker/). These plans included (1) virtual plans; (2) hybrid plans, where students divided time between being in-person and virtual; and (3) in-person plans. Hybrid return-to-learn plans differed in time spent in-person and virtually. Return-to-learn plans were implemented all over the world. Importantly, in a study conducted in Jordan, researchers found that most educators feared COVID-19 more than the potential ramifications of distance teaching.^12^

Before the pandemic, educators were at risk for depression and anxiety, with female sex and older age being associated with increased depression in educators.^13^ Educators describe the profession as physically, cognitively, emotionally, and socially stressful.^14^ Additionally, educators experience high levels of job-related stress when compared with the general public, and this stress was associated with poor job retention.^11,15^ Last, ongoing job-related stress is related to depression among educators.^16^ Depressive and anxiety symptoms are important to measure among educators during a global pandemic that is likely to amplify the related occupational stressors. This was further exemplified by a policy brief^17^ and technical report^18^ published by the American Psychological Association, which detailed that approximately half of US educators reported a desire or plan to leave or transfer jobs.

In addition to characterizing mental health symptoms during the pandemic, it is also important to identify modifiable factors that may impact mental health. One such factor is psychological flexibility, defined as the ability to mindfully engage in values-aligned behaviors even when experiencing difficult thoughts and emotions.^19^ The components of psychological flexibility, as specified in the triflex model, include openness to experiences, behavioral awareness, and valued action.^20^ *Openness to experiences* describes the willingness to feel unwanted internal experiences; *behavioral awareness* involves being present in the moment and closely observing the function of one’s behavior; and *valued action* emphasizes recognizing and acting in service of personally identified values.^21^ In the context of the COVID-19 pandemic, lower levels of psychological flexibility have been associated with higher levels of depression, anxiety, and stress, and higher levels of psychological flexibility have been associated with increased resilience.^22–24^ As psychological flexibility can act as a buffer against adverse outcomes following negative life events,^25,26^ it is plausible that psychological flexibility may be associated with psychiatric symptoms among educators during the COVID-19 pandemic.

## Objectives of the Current Study

Despite research suggesting that educators are at risk for depression, anxiety, and other psychiatric symptoms before the pandemic, COVID-19 presents a new context. Prior work has found that educators have experienced worsened mental health and well-being during the pandemic, but this work has not considered differing school reopening plans, nor has it accounted for county-level positive COVID-19 rates specific to geographic region. Considering the American Psychological Association (APA) calling for further empirical research regarding educators, investigations during the COVID-19 pandemic are particularly important. The present study investigated associations among 3 return-to-learn plans and internalizing (eg, depressive, social anxiety) symptoms in US educators, defined as teachers, paraeducators, administrators, and support staff, and how the components of psychological flexibility, a therapeutically modifiable process, relate to depressive and social anxiety symptoms. We hypothesized that teaching in-person, whether full-time in-person or hybrid, would be associated with greater depressive and social anxiety symptoms and that greater behavioral awareness, openness to experiences, and valued action would be associated with lower depressive and social anxiety symptoms.

## Methods

### Participants

Potential participants (*n* = 1783) began the survey with a screening, after which 726 entered the survey link but did not complete the survey, and 97 participants screened ineligible. Screening eligible included being an English-speaking adult living in the United States. Thus, 960 participants were eligible. After screening for valid responses to attention check items, 107 participants were excluded for invalid responses, and 853 valid respondents were included in the analyses (see Table 1 for demographic information).

### Procedures

Participants were recruited through Facebook and Reddit social media platforms and snowball sampling^27^ in November and December of 2020. Potential participants could click a link to the survey, which directed them to the survey site at qualtrics.com. Congruent with the institutional review board (IRB)-approved protocol, the first page of the survey was a consent letter notifying participants that clicking to the next page indicated consent to participate. The survey questions assessed demographic information, job characteristics, hardship during the COVID-19 pandemic, psychological symptoms, and psychological flexibility. Participants answered questions about the nature of work during the COVID-19 pandemic, school reopening plans, and public health precautions around COVID-19. This protocol was approved by the University of Iowa IRB, approval #202007406.

### Measures

#### Educational information

Survey questions examined the return-to-learn plans being used by educators’ school districts at the time of assessment. See Table 2 for a list of items that assessed job characteristics and return-to-learn plan information.

#### Depressive and anxiety symptoms

The Inventory of Depression and Anxiety Symptoms, version 2 (IDAS-II), is a 99-item measure developed to assess depressive and anxiety symptoms.^28^ The General Depression composite scale and the Social Anxiety subscale were used in congruence with prior research.^29,30^ Internal consistencies were adequate (see Table 3).

Clinical cutoffs corresponding to structured diagnostic interview data have been established for major depressive disorder.^31^ Normative data for United States adults^32^ were used to convert raw scores to *T*-scores. Norms for the measure were developed with adults dwelling in the United States before the COVID-19 pandemic.^32^ Quartiles of this sample’s *T*-scores are reported in Table 3.

#### Psychological flexibility

The Comprehensive Assessment of Acceptance and Commitment Therapy Processes (CompACT) is a 23-item validated measure of psychological flexibility components,^33^ including openness to experiences, behavioral awareness, and valued action. The CompACT is scored on a 7-point Likert scale ranging from 0 to 6, or “strongly disagree” to “strongly agree”, respectively. Higher scores indicate greater psychological flexibility. The measure demonstrated adequate reliability in this sample (total α: 0.90; openness to experience α: 0.84; behavioral awareness α: 0.82; valued action α: 0.84).

#### COVID-19 case rates by county

The Centers for Disease Control and Prevention (CDC) collected and reported publicly available positive case rates throughout the COVID-19 pandemic. The COVID-19 case rates were extracted from https://data.cdc.gov (dataset: “United States COVID-19 County Level of Community Transmission Historical Changes”). Data were extracted based upon the date of report, state, and county. Key COVID-19 indicators included: total number of new cases per 100,000 persons within the last 7 days, percentage of positive diagnostic and screening tests during the last 7 days, and the Community Transmission Level Indicator (low, moderate, substantial, high) from November and December of 2020. Notably, 173 participants did not have available COVID-19 data due to lack of available CDC data for the relevant date, or the participant did not provide state and county level data to match.

#### Statistical Analyses

IBM SPSS Statistics (Version 27) was used for analyses. Two one-way ANOVAs (analysis of variance) were used to examine between-group differences in return-to-learn plan (virtual, hybrid, in-person) as related to depressive and social anxiety symptoms. Two hierarchical linear regression models examined the association between psychological flexibility components and depressive and social anxiety symptoms. Demographic variables were examined for inclusion as covariates if associated with depressive or social anxiety symptoms. Continuous variables were examined with Pearson correlations, and dichotomous variables were examined with independent samples *t*-tests. County-level positivity rates for the past 7 days was included as a covariate in all analyses. Assumptions of homoscedasticity, normality, and independence were met. Missing data were minimal, and item-level missing data were imputed with person means by subscale if ≤20% of items were missing.^34^ There were 158 individuals missing CDC data in the social anxiety analyses and 173 individuals missing in the general depression analyses. As such, we conducted sensitivity analyses with and without the COVID-19 case rates, and results were comparable. Results reported herein are those with COVID-19 case rate data.

## Results

### Descriptive Characteristics

The sample was predominantly White (96.1%) and female-identifying (87.8%), with most educators being teachers (88.0%). Most educators (51.0%) reported a hybrid school reopening plan. Most educators (95.3%) reported having known someone who contracted COVID-19. Nearly all educators reported personally wearing a mask (99.2%) and social distancing (defined as keeping at least 6 feet of space between themselves and others) (95.4%). The majority of educators reported that social distancing guidelines were not enforced in the classroom (55.9%). Most educators reported face covering requirements on campus (93.5%). For more descriptive characteristics, see Table 1.

### Preliminary Analyses

There were negative correlations between age and depression *T*-scores (*r* = −.126; *n* = 758; *p* < 0.001) and social anxiety *T*-scores (*r* = −.202; *n* = 777; *p* < 0.001). Gender identity was examined categorically due to limited participants reporting transgender or genderqueer/gender-nonconforming identities. No significant differences were observed with regard to depression *T*-scores (*t*(822) = 0.61; *p* = 0.71) or social anxiety *T*-scores (*t*(843) = 1.22; *p* = 0.53) between female-identifying and male-identifying educators.

Established clinical cutoffs for depression were used as an indication of the clinical significance of reported depressive symptoms in this sample.^31^ The *screening* cutoff maximizes sensitivity, or the likelihood of correctly identifying someone with depression, and 64.8% of this sample fell above the screening threshold. The *balanced screening* cutoff balances sensitivity and specificity and identifies those experiencing mild or greater symptoms, and 40.0% of the current sample likely met criteria for at least mild symptoms of major depression. Finally, the *diagnostic* cutoff focuses on specificity, and 18.6% of this sample would likely be diagnosed with major depressive disorder.

Using established norms, *T*-scores were reported to contextualize educator mental health relative to a nationwide sample of US adults. As displayed in Table 3, median *T*-scores largely deviated from the normative mean.^32^ For example, the General Depression composite scale median was 81, which is 3 standard deviations above the normative mean. Traumatic intrusions were on average approximately 2.5 standard deviations above the normative mean. Irritability, or ill-temper, was nearly 3 standard deviations above the normative mean. See Table 3 for means, standard deviations, and *T*-score quartile values.

### Primary Analyses

#### General Depression

**Figure 1.**
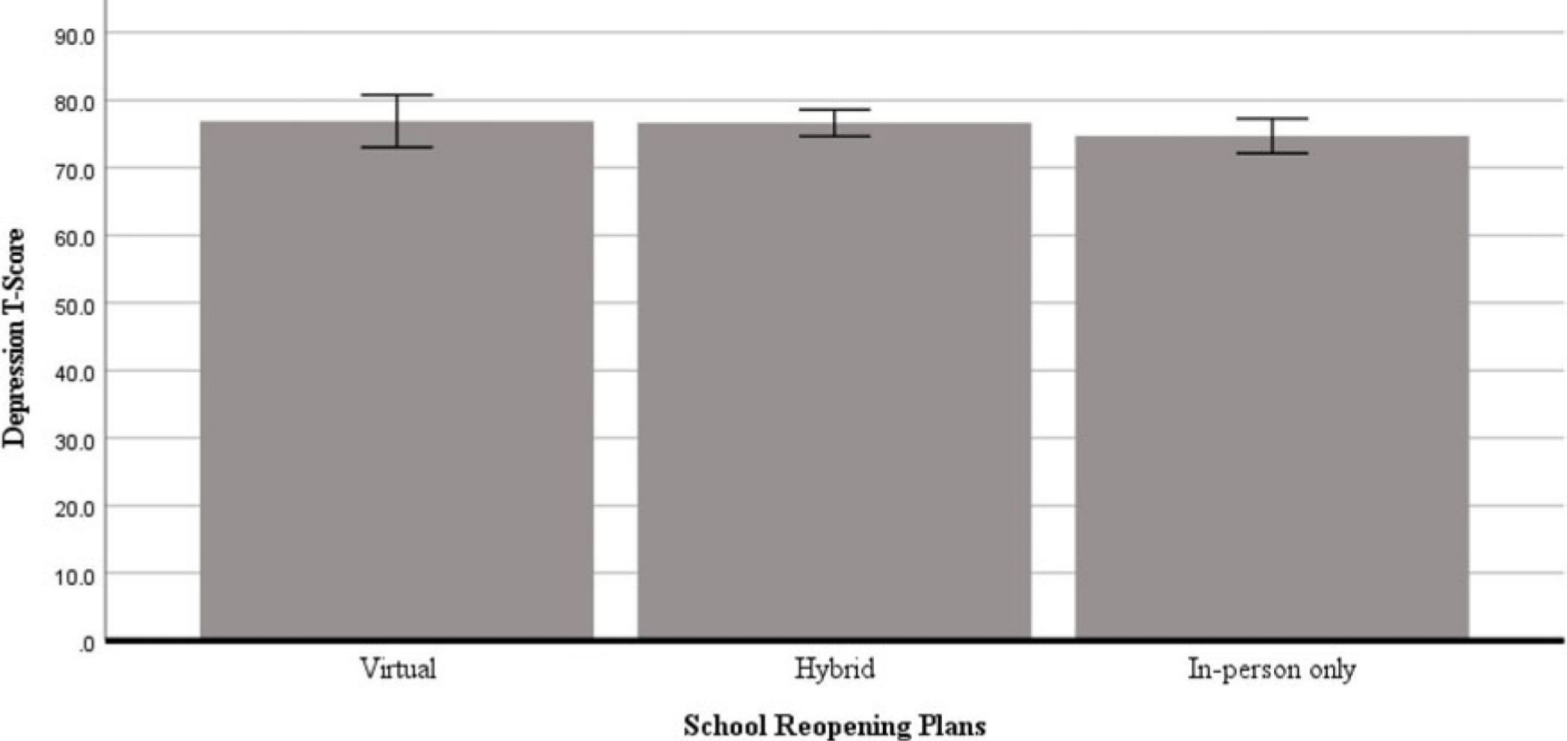
Between-group differences in depression across school reopening plans among US educators. Covariates appearing in the model are evaluated at the following values: Age = 42.73, Gender = .11, COVID-19 cases per 100k= 542.13. Error bars: +/− 1 SE.

There were no significant differences between reopening plans in general depression *T*-scores among educators, *F*(2, 680) = 0.77, *p* = 0.46, ηp^2^ = 0.002. Gender was not a significant covariate, *F*(1, 680) = 1.65, *p* = 0.20, ηp^2^ = 0.002, nor was COVID-19 county-level case rate, *F*(1, 680) = 0.82, *p* = 0.36, ηp^2^ = 0.001. Age was a significant covariate, *F*(1, 680) = 12.15, *p* < 0.001, ηp^2^ = 0.018. See Figure 1 for a graphical depiction of the findings.

The hierarchical regression model with depressive symptoms as the outcome included (1) age, gender, and COVID-19 positive rates as covariates; and (2) openness to experiences, behavioral awareness, and valued action as predictors. Results indicated that age, COVID-19 positivity rates, and gender were not significantly associated with depressive symptoms. Covariates accounted for 2% of the variance in depressive symptoms. Results indicated that behavioral awareness (B = −.98; *SE* = .12; β = −.33; *t*(664) = −8.17; *p* < 0.001) and openness to experiences (B = −.45; *SE* = .07; β = −.26; *t*(664) = −6.36; *p* < 0.001) were associated with lower depressive symptoms, whereas valued action was not significantly associated (B = −.18; *SE* = .10; β = −.06; *t*(664) = −1.71; *p* = .09). The final model (see Table 4) accounted for 33% (R^2^ = 0.33) of the variance in depressive symptoms.

#### Social Anxiety

**Figure 2.**
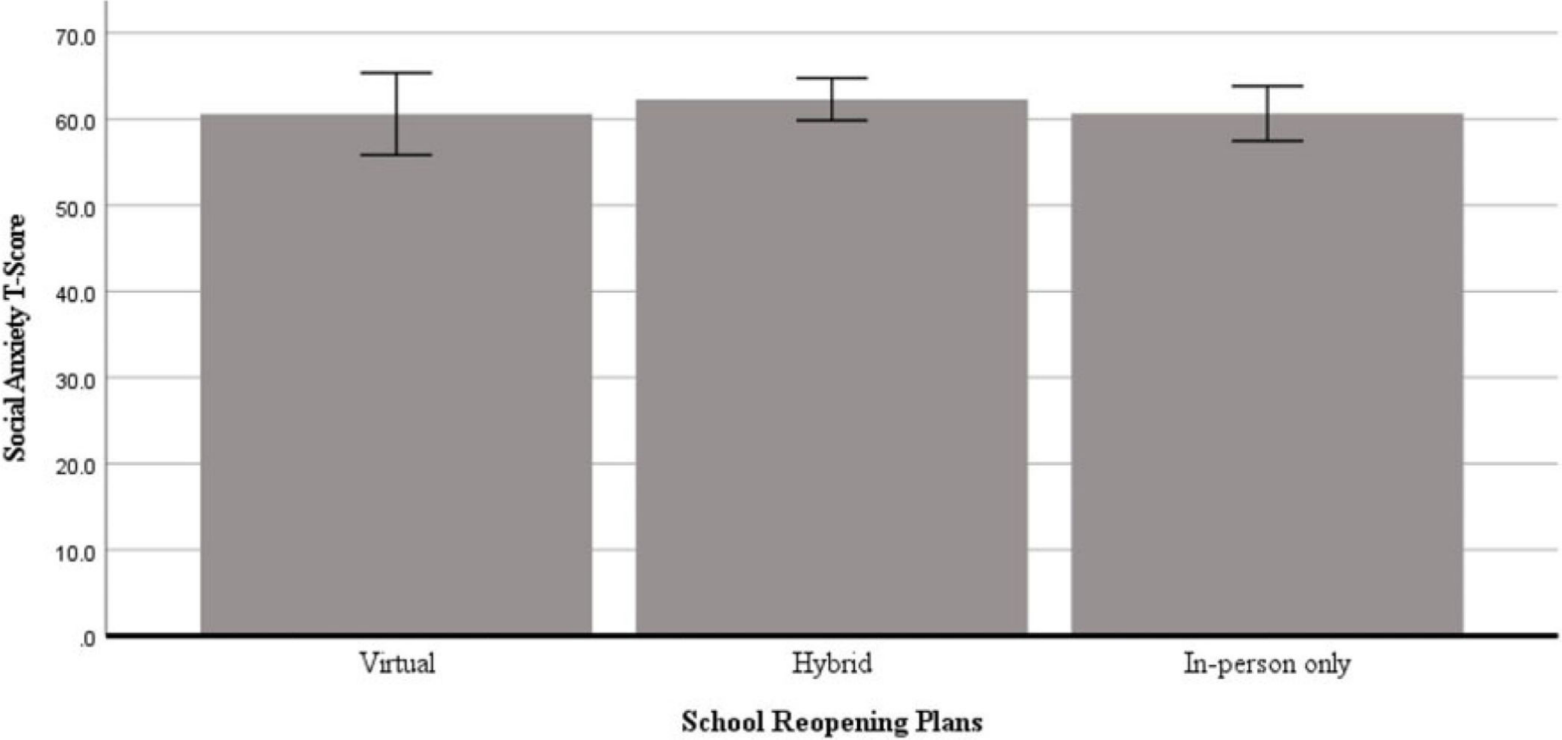
Between-group differences in social anxiety across school reopening plans among US educators. Covariates appearing in the model are evaluated at the following values: Age = 42.62, Gender = .11, COVID-19 cases per 100k = 540.02. Error bars: +/− 1 SE.

There were no significant differences between reopening plans in social anxiety *T*-scores among educators, *F*(2, 695) = 0.50, *p* = 0.61, ηp^2^ = 0.001. Gender was not a significant covariate, *F*(1, 695) = 0.09, *p* = 0.77, ηp^2^ = 0.000, nor was COVID-19 county-level case rate, *F*(1, 695) = 0.14, *p* = 0.71, ηp^2^ = 0.000. Age was a significant covariate, *F*(1, 695) = 27.21, *p* < 0.001, ηp^2^ = 0.04. The model, including age, gender, COVID-19 case rates, and reopening plan, did not account for substantial variance in social anxiety, R^2^ = 0.04. See graphical depiction of the findings in Figure 2.

The regression model evaluating social anxiety mirrored the prior model’s predictors and covariates. Results indicated that age was a significant covariate (B = −.25; *SE* = .07; β = −.12; *t*(678) = −3.42; *p* < .001), and COVID-19 positivity rates and gender were not significant. Covariates accounted for 4% of the variance in social anxiety symptoms. Results demonstrated that behavioral awareness (B = −.67; *SE* = .16; β = −.18; *t*(678) = −4.19; *p* < .001) and openness to experiences (B = −.63; *SE* = .10; β = −.29; *t*(678) = −6.56; *p* < .001) were associated with lower social anxiety, whereas valued action was not significantly associated (B = −.21; *SE* = .14; β = −.06; *t*(678) = −1.57; *p* = .12). The final model (see Table 5) accounted for 24% (R^2^ = 0.24) of the variance in social anxiety symptoms.

## Discussion

Amidst a global pandemic, educators were essential workers, and as schools transitioned into the fall 2020 semester, reopening plans differed across the United States. The goal of the present study was to investigate the effects of reopening plans on educator depressive and social anxiety symptoms during COVID-19 and to examine psychological flexibility’s relation with those symptoms. Data were collected in November and December of 2020, before availability of vaccinations in the United States, and during what was the highest peak of COVID-19 positive cases at that time^35^ (CDC, 2020). Perhaps most importantly, using prepandemic normative data collected from US adults,^32^ Depression, Social Anxiety, and most of the subscale *T*-scores demonstrated symptom elevations among our sample relative to normative data. The median *T*-score observed in General Depression, for example, was 3 standard deviations above the normative mean. Moreover, the median *T*-score for the well-being subscale was 2.5 standard deviations below the normative mean. Furthermore, clinical cutoffs indicated that over half of the sample would meet the most relaxed screening criteria for depression, and nearly one-fifth would likely meet diagnostic criteria for major depressive disorder. Finally, results indicated that there were no between-group differences in type of school reopening plan (virtual, hybrid, or in-person) as related to depressive and social anxiety symptoms among educators. Despite this lack of significant difference in type of school reopening plan, the alarmingly high rates of depressive and anxiety symptoms among educators warrant our attention. Additionally, these findings align with the APA technical and policy reports to indicate the importance of ongoing research with educators.^17,18^

Descriptively, the sample largely reported personal mask-wearing and adherence to social distancing recommendations. In addition, most educators endorsed knowing someone who had contracted COVID-19. Consequently, these variables could not be used for between-group comparisons given the small cell sizes between groups. Importantly, these descriptive findings help characterize the sample as a group that was broadly compliant with public health recommendations (e.g., mask wearing, social distancing), while also highlighting a discrepancy between personal behaviors and occupational context, particularly related to social distancing in the classroom. This finding is congruent with research demonstrating that healthcare professionals, also essential workers, were significantly more compliant with public health measures aimed at reducing the spread of COVID-19 when compared with non—healthcare professionals.^36^ Most of the sample also reported living in a county that had a “high” transmission level of COVID-19 cases, based on CDC data matched with survey completion date and county and state of residence. Age showed an inverse correlation with depressive and social anxiety *T*-scores, although the correlations were small in magnitude. Further research to characterize educator age, as well as years in the occupation, may help to elucidate these relationships. Openness to experiences and behavioral awareness were associated with lower depressive and social anxiety symptoms. These findings align with previous literature supporting psychological flexibility and mindfulness processes as key facilitators of reduced depressive and anxiety symptoms in treatment outcome research.^37–39^

The findings regarding elevated depressive and social anxiety symptoms are consistent with other research regarding the psychological impact of COVID-19.^4^ Symptoms of depression and anxiety among German residents and Spanish residents were both increased, and in Saudi Arabia, nearly one-fourth of participants reported a moderate to severe psychological impact of COVID-19, reflected in depression, anxiety, and stress.^40–42^ In the beginning stages of COVID-19 in the United States, psychological distress was considerably higher when compared with prepandemic distress.^43^ Global prevalence of depression and anxiety increased during COVID-19.^44^ This underscores the importance of screening for depression, anxiety, and other mental health symptoms during global disasters so that expeditious intervention can be provided.

Essential workers have experienced elevated distress and psychiatric symptoms during the COVID-19 pandemic. Physicians in India reported increased depression, anxiety, and stress, and medical and nursing staff in Wuhan, China, reported similar increases.^45,46^ In the United Kingdom, staff working on intensive care units reported substantial depression, anxiety, and psychological burden.^47^ Furthermore, in the United States, during November and December 2020, nationwide data showed a 13% increase in anxiety and depressive disorders.^48^ These findings show a consistent and pervasive increase in depression and anxiety during COVID-19.

### Future Directions and Implications

These results indicate a critical need to address depressive and anxiety symptoms in educators. Further characterization of the needs expressed by educators during the pandemic will be an important contribution to future intervention efforts. Aiding in this effort, qualitative data, in addition to the quantitative data presented herein, were collected, and these data are currently being examined to gain more nuanced insights into the impact of COVID-19 on educators. Moreover, most of this sample identified their educational role as “teacher”, although all educators were eligible. Future research should examine differences between educational role and mental health. Furthermore, in the APA technical report, which included qualitative data assessing the needs of educators, the inclusion of educator voices in decision-making was highlighted as necessary in reducing potential psychopathology and burden on educators.^17,18^ Investigation of potential mitigating factors, such as coping styles or social support is also important alongside focus on systems, policies, and practices.^49,50^ One modifiable factor measured in this study was psychological flexibility, or the ability to engage in values-aligned actions even in the presence of unwanted thoughts or emotions. Results suggest that future research should examine psychological flexibility interventions, such as Acceptance and Commitment Therapy (ACT^19^) for effectiveness with educators. ACT has demonstrated effectiveness across numerous outcomes, and changes in psychological flexibility were associated with improvements in physical and mental health outcomes.^19,51,52^ Importantly, despite promising findings about individual factors that are associated with decreased symptoms, it is critical that systemic and policy-level factors are addressed in tandem.

One solution to addressing treatment gaps (i.e., proportion of those who receive care relative to those who need care) is to integrate mental health supports, practices, programs, and policies into the workplace.^53,54^ The socio-political-economic environment shapes workplace conditions, which in turn affect employee health.^53,54^ Accounting for system-level and policy considerations in workplace settings, rather than solely focusing on individuals, has been shown to improve employee well-being in one study.^55^ Industrial-organizational psychologists have suggested altering scheduling practices and prioritizing essential tasks for employees who may be struggling.^56^ Additionally, employers, by contributing to healthcare costs and providing sick leave, have the capability to meet the increasing demand for mental healthcare services, which is crucial given the association between poor employee mental health, increased healthcare costs, and heightened use of sick time.^53^

Among individuals social distancing and isolating during COVID-19, social support mitigated depression and anxiety symptoms.^57,58^ Furthermore, social support is associated with increased psychological well-being.^59,60^ In focusing on social support, future research should also highlight the importance of community within the school system, as well as the broader community. Systems might focus on building community with the support of experts (e.g., industrial-organizational psychologists) who can develop destigmatizing mental healthcare interventions and a culture of professional-personal balance. Systemic support will provide the opportunity to seek mental healthcare, although individuals will have to choose to adopt these services to acquire the full benefit. Offering these resources may provide educators with agency to seek established and available services.

Another system-level priority is retention of educators, underscored by the APA’s 2022 report indicating a pervasive desire or plan to leave the profession.^17,18^ When teachers leave a school due to budget cuts or lack of funding, the resulting effects on student performance and school and district fiscal operations are “significant and deleterious.”^61,62^ Research has corrected the misconception that low teacher retention is due to limited student enrollment and/or teacher retirement; in actuality, teacher shortages are largely the result of teachers leaving schools or the profession before retirement.^61,62^ The cost of this is not only fiscal, but also emotional and psychological, with other teachers, students, and staff potentially facing negative impacts. The costs of teacher shortages also disproportionately impact schools that serve marginalized populations, including rural schools and students.^61,62^ As such, retention of teachers necessitates systemic and policy-level interventions. In addition to retention of educators in the profession, the APA’s 2022 report indicated that many teachers experienced violence from students and parents.^17^ Characterizing the broader community in which educators are functioning will be a critical component of future research.

A large proposed policy-level step is underway in the United States. In February of 2023, in the wake of the pandemic, the “Supporting the Mental Health of Educators and Staff Act of 2023” was introduced to the House of Representatives with bipartisan support and called for actionable steps aimed to address the observed decline in educator mental health.^63^ This proposed legislation arose following a survey in January of 2022, where nearly 75% of teachers and 85% of principals said they experienced frequent job-related stress, compared with one-third of non-educator working adults.^63^ The bill focuses on creating an initiative to promote mental health and substance use disorder services for educators aimed at destigmatizing mental health care, establishing federally funded programs for educator mental health care within the workplace, and requiring regular accountability-promoting reports of these programs’ efficacy.^63^

Mindfulness-based interventions have been examined with educators and may be a candidate for integration into the workplace for efficacy examination. Mindfulness-based interventions aim to increase awareness and well-being, which have demonstrated benefit in the workplace, and focus on increasing positive outcomes in lieu of the “deficit model” or reduction of negative outcomes.^14,64,65^ Workshops focused on building social-emotional competencies, including mindfulness practice, were found to benefit educators across eight different schools in Colorado.^66^ Among female teachers in Italy during COVID-19, individuals who received a mindfulness intervention experienced improvements in depressive symptoms and psychological well-being compared with the control group.^67^ As such, mindfulness-based interventions tailored to the educator experience could improve educator mental health and warrant further investigation. In prioritizing the mental health of educators, counselors for teachers in schools, separate from the school psychologist that supports students, have been found to improve mindfulness and decrease stress.^68^ Furthermore, conducting mental health screenings when doing primary healthcare visits has been effective.^69^ Within the workplace, mental health screenings of educators could also be beneficial for those needing additional support or workplace-supported intervention.

Given ongoing global disasters, studying educator mental health and the impact of disasters on educator mental health is of paramount importance. While all individuals experiencing disasters may be impacted, educators may be uniquely affected professionally. Characterizing these occupational demands is important in each context to provide adequate resources. Moreover, educators may experience vicarious stress by way of their students, and trauma-informed principles may be appropriate in terms of training in disaster preparedness (e.g., Psychological First Aid).^70^ Characterizing the experience of educators during collective stressors will be important in future research given their critical role in impacting communities and the known mental health risks associated with this profession.

### Limitations

These findings should be considered with several limitations in mind. Our study predominantly identified as female and White, despite our efforts to recruit in educator groups on Facebook and Reddit that represented educators from all 50 states, as well as people of all genders, ages, races, and ethnicities. The sample was also limited to those with access to social media or personal contact with social media users. As such, generalizability of these results is limited. However, it should be noted that, in larger studies of educators, sample homogeneity was also observed, with the APA conducting a study among United States educators that was 81% female-identifying and 77% White.^17^ Additionally, a demographic breakdown of teachers at the K-12 level in the United States from 2017 to 2018 found that teachers were 79% White and 76% female-identifying (National Center for Education Statistics: US Department of Education, 2018).^71^ Furthermore, this was a cross-sectional assessment of each educator’s experience, and occupational circumstances may have changed before or after survey participation. This was a cross-sectional study, so no causal inferences can be made. Because we did not collect depression and/or anxiety data before COVID-19, no longitudinal changes were observed.

## Conclusions

The COVID-19 pandemic has adversely impacted physical and mental health, and educators were no exception. Although there is substantial literature investigating the impact of COVID-19 on essential workers (e.g., medical providers), there is limited literature regarding educators specifically. This study aimed to examine depressive and anxiety symptoms among educators during COVID-19 and investigate return-to-learn plan as a potential variable of interest. Despite a lack of between-group differences in return-to-learn plans, depression and social anxiety were elevated compared with normative data. Indicators of clinical significance suggested high rates of depressive symptoms among our sample. Beyond the COVID-19 pandemic, educators will continue to navigate global and local stressors in the classroom and play a critical role in the development of youth and communities. As the world looks toward future disasters, it is essential that lessons learned during COVID-19 be preserved. The results provide important information about future screening efforts and possible areas for intervention across individuals, systems, policies, and practices. In conclusion, future intervention efforts should prioritize educators as a group who have been impacted substantially by the the COVID-19 pandemic.

## Figures and Tables

**Table 1.** Descriptive characteristics of the sample, N = 853

Parameter	n (%)
Age, *M*(SD)	42.65 (11.3)
Personal income^a^	
$0 through $49,999	282 (33.3%)
$50,000 through $74,999	408 (48.0%)
$75,000 and greater	128 (15.1%)
Missing	35 (3.7%)
Race	
White	820 (96.1%)
African American or Black	3 (0.4%)
American Indian or Alaska Native	5 (0.6%)
Asian	5 (0.6%)
Biracial or Multiracial	15 (1.8%)
Native Hawaiian or Pacific Islander	1 (0.1%)
Did not disclose	4 (0.5%)
Ethnicity	
Non-Hispanic	823 (96.5%)
Hispanic	19 (2.2%)
Did not disclose	11 (1.3%)
Gender identity	
Female	749 (87.8%)
Male	95 (11.1%)
Transgender man	1 (0.1%)
Genderqueer/gender-nonconforming	6 (0.7%)
Prefer to self-describe	1 (0.1%)
Prefer not to disclose	1 (0.1%)
Role in school system	
Administrator	70 (8.2%)
Teacher	751 (88.0%)
Paraeducator	32 (3.8%)
Grade level taught by teachers^b^	
Pre-kindergarten	45 (5.9%)
Elementary	297 (38.9%)
Middle school	199 (26.1%)
High school	295 (38.6%)
College	33 (4.3%)
Reopening plans	
Remote learning only	131 (15.4%)
Hybrid	432 (50.6%)
Full-time in-person	290 (34.0%)
COVID-19 community transmission level	
High	757 (88.7%)
Substantial	11 (1.3%)
Moderate	4 (0.5%)
Low	0 (0.0%)
Missing	81 (9.5%)
COVID-19 cases per 100k, M(SD), *N* = 771	559.56 (362.18)
Percent of COVID-19 positive tests in past 7 days, M(SD), *N* = 772	15.48 (7.44)

a Data were consolidated for descriptive purposes.

b Participants could select multiple options if applicable.

**Table 2.** Questions relating to educator experiences and COVID-19

Question	Response options
What is your role within the school system?	Administrator or support staff Teacher Paraeducator
What plan most closely resembles the plan that your district or school has determined to reopen your school during the COVID-19 pandemic?	Virtual learning Hybrid plan Full-time in-person
Did you know someone that contracted COVID-19?	Yes No
Do you personally wear a mask to help slow the spread of COVID-19?	Yes No
Are face coverings required when students are on-campus/in the school?	Yes No
Are appropriate face coverings provided for you and/or students who do not have them?	Yes No
Are social distancing guidelines enforced in the classroom?	Yes No
Were you given the opportunity to request remote work options, regardless of whether or not you chose to do so?	Yes No

**Table 3.** Depressive and Anxiety subscale *T*-score means, standard deviations, quartile scores, and internal consistencies (N = 853)

IDAS-II subscale	α	Mean	SD	25^th^ %ile	50^th^ %ile	75^th^ %ile
General Depression	0.88	76.18	18.83	67	81	91
Dysphoria	0.89	75.63	19.44	67	79	90
Lassitude	0.80	71.76	21.66	60	75	88
Insomnia	0.87	69.03	24.56	54	75	90
Suicidality	0.78	48.48	25.32	25	25	74
Appetite Loss	0.86	67.93	16.06	50	67	83
Appetite Gain	0.83	65.31	25.90	48	71	85
Well-being	0.88	27.42	20.74	11	24	40
Ill-temper	0.87	70.59	23.95	51	78	88
Mania	0.85	58.29	27.27	41	60	82
Euphoria	0.65	29.42	21.88	10	27	39
Panic	0.83	63.34	24.53	52	71	83.5
Social Anxiety	0.80	61.77	23.88	42	67	82
Claustrophobia	0.86	62.46	27.15	32	74	87
Traumatic Intrusions	0.81	67.18	23.30	57	74	84
Traumatic Avoidance	0.85	48.73	26.63	16	47	72
Checking	0.85	48.94	29.07	13	52	75
Ordering	0.82	50.76	27.55	14	49	76
Cleaning	0.86	73.09	24.76	65	81	92

*Note:* IDAS-II, Inventory of Depression and Anxiety Symptoms, second edition. α, internal consistency; %ile, percentile.

**Table 4.** Components of psychological flexibility as associated with depressive symptoms

Step	B	SE	β	*t*	*p*	CI for B	R^2^
Step 1							.02
COVID-19 case rate	.01	.10	.01	.13	.89	−.18, .21	
Age	−.26	.06	−.15	−3.98	<.001	−.38, −.13	
Gender identity	−2.01	2.35	−.03	−.85	.39	−6.62, 2.61	
Final model - Steps 1 and 2							0.33
COVID-19 case rate	.06	.08	.02	.71	.48	−.10, .22	
Age	−.09	.05	−.06	−1.71	.09	−.20, .01	
Gender identity	−2.04	1.95	−.03	−1.04	.30	−5.87, 1.80	
**Openness to experiences**	−.45	.07	−.26	−6.36	<.001	−.59, −.31	
**Behavioral awareness**	−.98	.12	−.33	−8.17	<.001	−1.21, −.74	
Valued action	−.18	.10	−.06	−1.71	.09	−.38, .03	

*Note:* Gender identity was coded 0 = female-identifying, 1 = male-identifying. Openness to experiences, behavioral awareness, and valued action were measured using the Comprehensive Assessment of Acceptance and Commitment Therapy processes. Bolded rows signify statistically significant findings.

**Table 5.** Components of psychological flexibility as associated with social anxiety symptoms

Step	B	SE	Beta	*t*	*p*	CI for B	R^2^
Step 1							.04
COVID-19 case rate	−.03	.12	−.01	−.20	.84	−.27, .22	
Age	−.42	.08	−.20	−5.32	<.001	−.58, −.27	
Gender identity	−4.81	2.91	−.06	−1.65	.10	−10.53, .91	
Final model - Steps 1 and 2							.24
COVID-19 case rate	.01	.11	.00	.09	.93	−.21, .23	
**Age**	**−.25**	**.07**	**−.12**	**−3.42**	**<.001**	**−.39, −.11**	
Gender identity	−4.59	2.60	−.06	−1.77	.08	−9.69, .51	
**Openness to experiences**	**−.63**	**.10**	**−.29**	**−6.56**	**<.001**	**−.81, −.44**	
**Behavioral awareness**	**−.67**	**.16**	**−.18**	**−4.19**	**<.001**	**−.98, −.36**	
Valued action	−.21	.14	−.06	−1.57	.12	−.48, .05	

*Note:* Gender identity was coded 0 = female-identifying, 1 = male-identifying. Openness to experiences, behavioral awareness, and valued action were measured using the Comprehensive Assessment of Acceptance and Commitment Therapy processes. Bolded rows signify statistically significant findings.

## Data Availability

Deidentified or aggregated data will be made available upon reasonable request, congruent with the approved IRB application.
